# Common cancer-associated imbalances in the DNA damage response confer sensitivity to single agent ATR inhibition

**DOI:** 10.18632/oncotarget.6136

**Published:** 2015-10-15

**Authors:** Fiona K. Middleton, Miranda J. Patterson, Claire J. Elstob, Sarah Fordham, Ashleigh Herriott, Mark A. Wade, Aiste McCormick, Richard Edmondson, Felicity E.B. May, James M. Allan, John R. Pollard, Nicola J. Curtin

**Affiliations:** ^1^ Newcastle University, Northern Institute for Cancer Research, Newcastle upon Tyne, UK; ^2^ Vertex Pharmaceuticals (Europe) Limited, Milton Park, Abingdon, Oxfordshire, UK

**Keywords:** ATR, p53, DNA damage response, DNA-PKcs, synthetic lethality

## Abstract

ATRis an attractive target in cancer therapy because it signals replication stress and DNA lesions for repair and to S/G2 checkpoints. Cancer-specific defects in the DNA damage response (DDR) may render cancer cells vulnerable to ATR inhibition alone. We determined the cytotoxicity of the ATR inhibitor VE-821 in isogenically matched cells with DDR imbalance. Cell cycle arrest, DNA damage accumulation and repair were determined following VE-821 exposure.

Defectsin homologous recombination repair (HRR: ATM, BRCA2 and XRCC3) and baseexcision repair (BER: XRCC1) conferred sensitivity to VE-821. Surprisingly, the loss of different components of the trimeric non-homologous end-joining (NHEJ) protein DNA-PK had opposing effects. Loss of the DNA-binding component, Ku80, caused hypersensitivity to VE-821, but loss of its partner catalytic subunit, DNA-PKcs, did not. Unexpectedly, VE-821 was particularly cytotoxic to human and hamster cells expressing high levels of DNA-PKcs. High DNA-PKcs was associated with replicative stress and activation of the DDR. VE-821 suppressed HRR, determined by RAD51 focus formation, to a greater extent in cells with high DNA-PKcs.

Defects in HRR and BER and high DNA-PKcs expression, that are common in cancer, confer sensitivity to ATR inhibitor monotherapy and may be developed as predictive biomarkers for personalised medicine.

## INTRODUCTION

The DNA damage response (DDR) is essential to maintain genomic stability in the face of the high rate of ongoing insult from environmental and endogenous sources of DNA damage [1]. The DDR signals DNA damage for repair, and to cell cycle checkpoints that arrest the cell cycle while repair is being completed. The response to DNA double strand breaks (DSBs) and collapsed replication forks is particularly crucial as these types of damage are difficult to repair. Three PI3-Kinase-related kinases (PIKKs) ATM (ataxia telangiectasia mutated), ATR (ATM and Rad3 related) and DNA-PKcs (DNA-dependent protein kinase catalytic subunit) recruit DNA repair proteins and activate cell cycle checkpoints in response to these lesions [2]. ATR is arguably the most versatile of these three PIKKs because it is activated by regions of single-stranded DNA that occur during nucleotide excision repair, following resection of DNA DSBs and, lesions most critical to replicating cells, collapsed replication forks. Activated ATR phosphorylates a number of targets involved in homologous recombination DNA repair (HRR) and the re-start of replication forks but its major target is CHK1 (reviewed in Chen et al. [3]). By phosphorylation of CHK1, ATR initiates the S and G2 checkpoint cascade.

Aberrations in the DDR create the genomic instability that is an “enabling characteristic” of cancer [4]. Cancer cells commonly have dysregulated G1 cell cycle arrest, e.g. due to inactivation of the TP53/RB pathway [5], and rely on their S/G2 checkpoints for survival following DNA damage. Exploiting this dependence makes the ATR/CHK1 axis an attractive target to selectively enhance the anti-cancer activity of DNA damaging chemotherapy and radiotherapy. CHK1 inhibitors, and more recently ATR inhibitors attenuate/abrogate cell cycle arrest and increase the cytotoxicity of the major classes of DNA damaging anticancer agents preclinically (reviewed in [3]). CHK1 inhibitors are yet to fulfil their pre-clinical promise in clinical trials, possibly because of poor selectivity resulting in toxicity in combination (reviewed in [3]). Preclinically ATR inhibition has somewhat different effects from CHK1 inhibition [6] and if inhibitors could be used as single agents toxicities are expected to be minimal [7]. Two ATR inhibitors, VX-970 and AZD-6738, are currently undergoing clinical evaluation. VX-970 is being evaluated both as a single agent and in combination with platinum or gemcitabine chemotherapy (clinicaltrials.gov identifier: NCT02157792) and AZD-6738 in combination with ionising radiation and carboplatin (EudraCT identifier: 2013-005100-34 and clinicaltrials.gov identifier: NCT02223923 and NCT02264678).

Exploitation of the dysregulation of the DDR by synthetic lethality through the single-agent use of inhibitors of other DDR components, exemplified by the cytotoxicity of PARP inhibitors in HRR-defective tumours [8] is an exciting new paradigm in cancer that led to a rapid expansion in PARP inhibitor development. This has driven the search for other synthetic lethal interactions that could be exploited therapeutically. Recent studies with the prototype ATR inhibitor, NU6027, demonstrated that it was particularly cytotoxic to cells defective in BER (XRCC1 mutant), and synergistic with PARP inhibition [9, 10]. ATM and ERCC1-XPF defects are also reported to render cells more sensitive to ATR inhibition [11]. We therefore sought to identify DDR defects synthetically lethal with the ATR inhibitor, VE-821 (the pre-clinical lead from which VX-970 was developed), as a single agent and to explore the underlying mechanism.

We confirm that cells lacking ATM or the BER scaffold protein, XRCC1, are hypersensitive to ATR inhibitor-induced cytotoxicity and identify for the first time that defects in HRR components, XRCC3 or BRCA2, also confer sensitivity to ATR inhibition. Interestingly, while loss of the binding partner of DNA-PKcs, XRCC5 (Ku80), conferred sensitivity to VE-821, loss of DNA-PKcs itself conferred resistance and over-expression of DNA-PKcs conferred sensitivity. Mechanistic studies suggest this is due to increased replicative stress and attenuation of HRR function.

## RESULTS

### VE-821-induced cytotoxicity in DNA repair defective Chinese hamster cells

**Figure 1:**
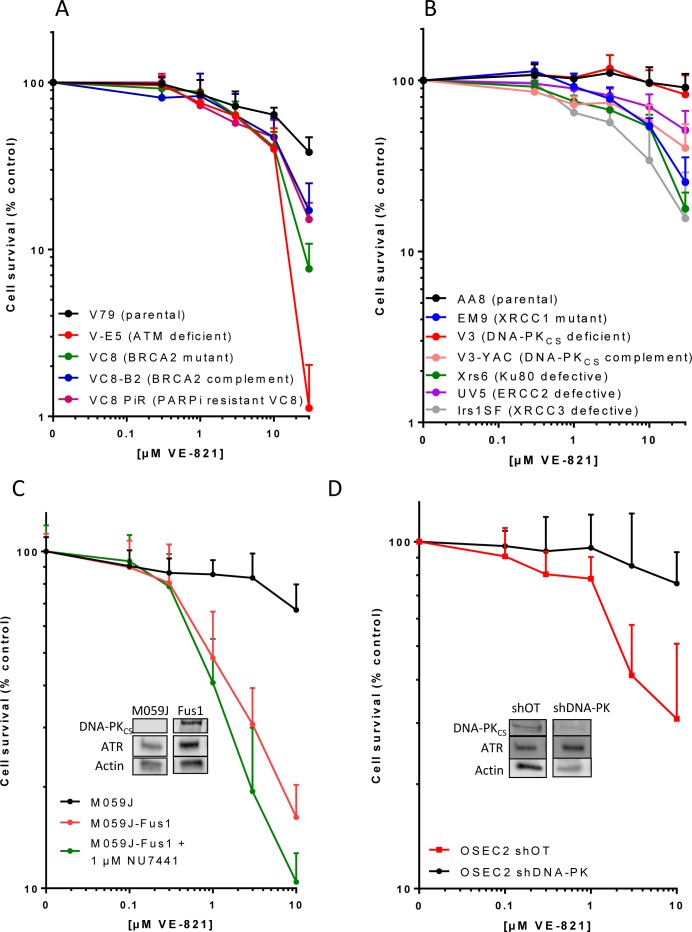
The cytotoxicity of single-agent VE-821 in cells with different DDR defects Cells were exposed to varying concentrations of VE-821 for 24 hr then allowed to form colonies in drug free medium. Data are mean and standard deviation of 3 independent experiments for **A.** Chinese hamster lung cells: V79 (parental), V-E5 (ATM mutant, checkpoint deficient), V-C8 (BRCA2 mutant, HRR defective), V-C8 B2 (V-C8 cells complemented with wt BRCA2) and V-C8 PiR (PARPi-resistant V-C8 with secondary mutation in BRCA2 restoring function), **B.** Chinese hamster ovary cells: AA8 (parental wt), EM9 (XRCC1 mutant, BER defective), V3 (DNA-PKcs mutant, NHEJ defective), V3-YAC (DNA-PKcs restored with yeast artificial chromosome), Xrs6 (Ku80 mutant, NHEJ defective), UV5 (ERCC2 mutant, NER defective), Irs1SF (XRCC3 mutant, HRR defective), **C.** Human glioma cells M059J (DNA-PKcs deficient), M059J-Fus1 (DNA-PKcs corrected by transfer of part of Chromosome 8) and M059J-Fus1 co-exposed to the DNA-PK inhibitor, NU7441 (1 μM), **D.** Human ovarian cancer cells OSEC2 shDNA-PK (with DNA-PKcs knockdown) and OSEC2 shOT (off target knockdown). Inserts in C and D show levels of DNA-PKcs and ATR in the cells.

In previous studies with VE-821 the greatest chemosensitisation was observed in cells lacking ATM [7] and VE-821 was synthetically lethal in cells depleted of ATM [12]. We show here that V-E5 cells lacking ATM are the most sensitive to VE-821 alone with only 1% surviving exposure to 30 μM VE-821 compared to 38% in the parental V79 Chinese hamster lung cells (*p* = 0.01) (Figure 1A, Table 1). V-C8 cells that are HRR defective, by virtue of a BRCA2 mutation, were almost as sensitive (8% survival *p* = 0.04). Restoring BRCA2 function through transfection of wt BRCA2 (V-C8 B2) or through a reversing mutation (V-C8 PiR) resulted in reduced sensitivity to VE-821.

Chinese hamster ovary AA8 cells were intrinsically resistant to single agent VE-821 with 30 μM having virtually no impact on viability (Figure 1B). This was not due to a failure of ATR inhibition because VE-821 reduced pChk1s^345^ to a similar or greater extent in AA8 cell lines compared to V79 cells and M059J cells (Supplementary Figure S1). EM9 cells lacking BER function due to XRCC1 loss were significantly (*p* < 0.0001) more sensitive to VE-821 with 30 μM killing approximately 75% (Table 1). The HRR-defective Irs1SF (XRCC3 mutant) were the most sensitive of the AA8 derivatives with only 16% surviving exposure to 30 μM VE-821. The UV5 cells that are nucleotide excision repair defective due to ERCC2 mutation were also significantly (*p* = 0.0002) more sensitive than the parental cells, but were the least sensitive of all the repair-defective CHO cells. Most curious was the data with non-homologous end joining (NHEJ) defective cells. Ku70 and Ku80 bind DNA DSB and recruit DNA-PKcs to form the catalytically active holoenzyme to promote DSB repair. Ku80-defective xrs6 cells showed sensitivity comparable with HRR and BER defective cells but, surprisingly, the V3 cells, defective in DNA-PKcs, were not hypersensitive to VE-821 (Figure 1B, Table 1). Correction of the DNA-PKcs defect by transfection of a YAC containing human DNA-PKcs rendered the cells (V3-YAC) significantly (*p* < 0.0001) more sensitive to VE-821 (only 40% survival at 30 μM).

**Table 1:** VE-821 cytotoxicity in cell lines with differing DDR status

Species and tissue of origin	Cell line	DNA repair defect/pathway	Significant difference from parental* *p*=	% Survival at 10 uM VE-821	% Survival at 30 uM VE-821
Chinese hamster ovary	AA8	Parental wt		97 ± 22†	90.8 ± 18.3
	EM9	XRCC1BER	*p*<0.0001	54 ± 6	25.4 ± 10.1
	UV5	ERCC2/NER	*p* = 0.0002	70 ± 13	51.1 ± 15.2
	Irs-1SF	XRCC3/HRR	*p*<0.0001	34 ± 20	15.6 ± 13.3
	Xrs6	Ku80/NHEJ	*p*<0.0001	53 ± 10	17.8 ± 4.3
	V3	DNA-PKcs/NHEJ	*p* = 0.9966	96 ± 18	82.6 ± 24.5
	V3-YAC	Corrected V3	*p*<0.0001	41 ± 9	40.3 ± 15.2
					
Chinese hamster lung	V79	Parental wt		64 ± 7	38.3 ± 8.7
	V-E5	ATM/cell cycle checkpoint	*p* = 0.01	40 ± 13	1.1 ± 0.9
	V-C8	BRCA2/HRR	*p* = 0.04	41 ± 9	7.7 ± 3.2
	V-C8 B2	BRCA2 corrected	*p* = 0.17	47 ±14	17.1 ± 7.8
	V-C8 PiR	BRCA2 revertant	*p* = 0.03	47 ± 13	15.1 ± 3.9
					
Human GBM	M059J	DNA-PKcs/NHEJ		67 ± 13	
	M059-Fus-1	DNA-PKcs corrected	*p*<0.00001	16 ± 4	
	M059-Fus-1 + NU7441	DNA-PKcs corrected + inhibited	*p*<0.00001	10 ± 2	
					
Human ovarian surface epithelium	OSEC2 shOT	parental		31 ± 20	
	OSEC2 shDNA-PKcs	DNA-PKcs/NHEJ	*p* = 0.0023	76 ± 17	

* Statistical differences between cell sensitivities were calculated using a 2-way ANOVA and the p values shown.

† Data are mean ± standard deviation of the % survival at 10 μM VE-821.

### VE-821-induced cytotoxicity in human cells with high levels of DNA-PKcs

Because of the unexpected results with the Chinese hamster DNA-PKcs proficient and deficient cells we investigated the phenomenon further in human malignant glioblastoma cells deficient in DNA-PKcs, M059J, and the DNA-PKcs overexpressing M059J-Fus-1 cells (hereafter called Fus-1 cells for simplicity) (Figure 1C). Fus-1 cells were substantially and significantly (*p* < 0.0001) more sensitive to VE-821 with only 16% surviving treatment with 10 μM in comparison with the DNA-PK defective M059J cells with 67% survival. To determine if DNA-PKcs kinase activity was responsible we used NU7441, a potent and specific DNA-PK inhibitor [13], at a concentration of 1 μM (as previously used for chemo- and radiosensitisation and approximately 5x the cellular IC_50_ [14]). Co-exposure of the M059J Fus-1 cells to NU7441 did not protect from VE-821 cytotoxicity, in fact it increased cell kill (10% survival at 10 μM VE-821; Table 1; Figure 1C). This was not due to an off-target effect because NU7441 failed to sensitise M059J cells to VE-821 (Supplementary Figure S3) Further investigations in human ovarian OSEC2 cells (selected because of a high intrinsic level of DNA-PKcs with an efficient knockdown: A McCormick, unpublished data) revealed that 91% DNA-PKcs knockdown resulted in significant protection from VE-821 cytotoxicity (Figure 1D, Table 1). Thus, a consistent pattern of greater sensitivity of high DNA-PKcs expressing cells to VE-821 was seen in 3 independent cell line pairs. Differences in sensitivity to VE-821 were unlikely to be due to different ATR expression levels since ATR levels were equivalent or slightly higher in OSEC2 shDNA-PKcs cells (Figure 1D insert), but lower in the DNA-PKcs deficient M059J cells (Figure 1C insert) when normalised to actin loading control compared to their DNA-PKcs expressing counterparts. The greater sensitivity of the DNA-PKcs expressing cells was also not due to greater inhibition of ATR activity by VE-821 asVE-821 (10 μM) inhibited CHK1^Ser345^ phosphorylation to a similar extent in both M059J and Fus-1 cells (Supplementary Figures S1 and S2). *These data suggest that the abundance of DNA-PK_CS_ protein, independent of its kinase activity, sensitises cells to VE-821-induced cytotoxicity.*

**Figure 2:**
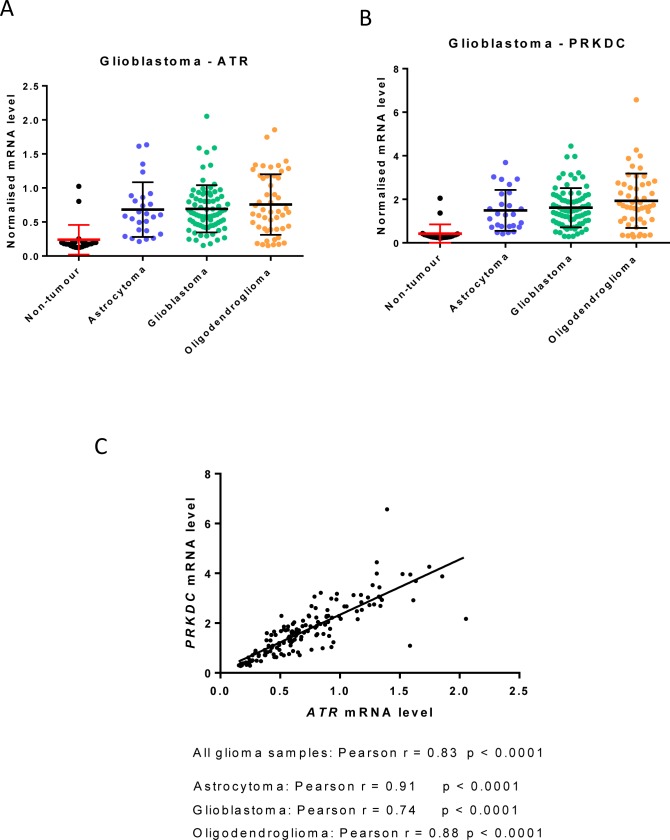
ATR and PRKDC (DNA-PKcs) mRNA expression in glioblastoma subtypes Data are from publically available NCBI Gene Expression Omnibus (GEO) dataset GSE4290 and mean *ATR* and *PRKDC* mRNA were normalised to *HPRT1*. Samples were from 23 non-tumour brain (epilepsy patients), 26 astrocytomas, 77 glioblastomas and 50 oligodendrogliomas. **A.** *ATR* **B.** *PRKDC* **C.** Correlation of *ATR* and *PRKDC* mRNA expression using GraphPad Prism version 6.0.

Interestingly, mining of publically-available data suggests that the expression of ATR (*ATR*) and DNA-PKcs (*PRKDC*) may be tandemly regulated in certain tumours. For example, the expression of *ATR* and *PRKDC* are similarly elevated across diverse glioblastoma subtypes compared to normal brain (Figure 2A and 2B) and their expression is strongly correlated (Figure 2C).

### cMYC protein levels depend on DNA-PKcs

**Figure 3:**
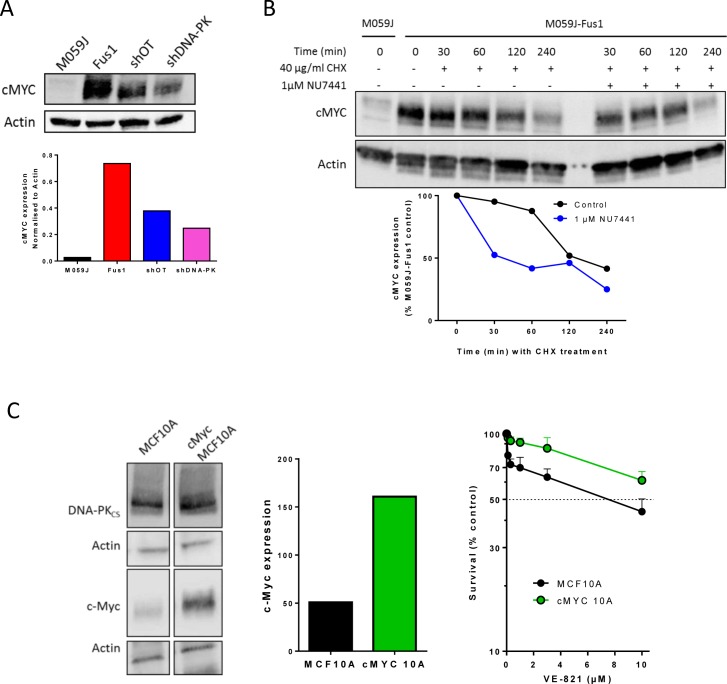
The interaction between cMyc and DNA-PKcs and the cytotoxicity of VE-821 **A.** cMYC expression in M059J, M059J-Fus-1, OSEC2 shOT and OSEC2 shDNA-PK cells, upper panel representative Western blot lower panel, data normalised to actin. Data are from a single experiment, similar data are shown in Supplementary Figure S4. **B.** Impact of DNA-PK inhibitor on cMYC stability. M059J-Fus-1 cells were exposed (or not) to NU7441 (1 μM) to inhibit DNA-PK and/or cyclohexamide (CHX) to inhibit new protein synthesis for the times shown prior to protein extraction and measurement by Western blot, upper panel representative Western blot lower panel, data normalised to zero time untreated control. Data are from a single experiment at these time-points. **C.** DNA-PKcs and cMYC levels measured By Western blot (left panel), normalised to Actin loading control (centre panel) and the cytotoxicity of VE-821 in human breast epithelial cells, MCF10A and its derivative cMYC-MCF10A (cMYC10A). Data are from a single experiment, with expression data confirming data published in [1].

Amplified cMYC may confer replicative stress and sensitivity to inhibitors of both ATR and CHK1 [15]. Fus-1 cells contain 7 or 8 copies of chromosome 8 following transfection to correct their DNA-PKCS defect (encoded by *PRKDC* located at 8q11.21) [16] we assessed levels of cMYC (also located on chromosome 8 at 8q24.21) in both M059J and Fus-1 cells. Despite containing 3 or 4 copies of chromosome 8 [17] M059J cells expressed only low levels of cMYC whereas Fus-1 cells expressed much higher cMYC levels (Figure 3A, Supplementary Figure S4). DNA-PKcs may be necessary to stabilise cMYC [18] so we determined cMYC levels in OSEC2 cells with (shDNA-PK) and without (shOT) silenced DNA-PKcs (Figure 3A, Supplementary Figure S4) and again cMYC levels were lower in cells with reduced DNA-PKcs expression. Therefore the higher levels of cMYC in the Fus1 cells and OSEC2 cells could have affected VE821 sensitivity.

To determine whether DNA-PK activity was required to maintain high cMYC levels we investigated the effect of NU7441 (1 μM to allow direct comparison with the cytotoxicity data) on cMYC levels in Fus-1 cells. cMYC is reported to have a short half-life of 20-30 min [19]. In our study the levels of cMYC declined less rapidly in Fus-1 cells with an estimated half-life of approximately 2 hours, suggesting some stabilisation. In NU7441-treated cells the decline was more rapid and cMYC levels were reduced to 50% of the initial level at 30 min (Figure 3B). Since NU7441 impaired cMYC stability (Figure 3B) but did not protect from VE-821 cytotoxicity, sensitivity to VE-821 may not be due to elevated cMYC. To test this hypothesis further we used immortalised human breast cells, MCF10A and a *MYC*-amplified derivative (cMYC-MCF10A) with an approximately 3-fold higher level of cMYC expression. In cytotoxicity assays the *MYC*-amplified cells were more resistant rather than more sensitive to VE-821 compared to parental cells (Figure 3C; p < 0.0001). Taken together these data suggest that sensitivity to VE-821 is not due to elevated cMYC.

### VE-821 impairs HRR in irradiated DNA-PKcs overexpressing cells

We next investigated if the preferential cytotoxicity of VE-821 in cells with high DNA-PKcs levels was due to an effect on DNA repair. DNA is continuously being damaged at an astonishingly high level (>10^5^ SSBs/genome/cell/day) largely caused by reactive oxygen species that are a by-product of respiration. In replicating cells, any remaining un-repaired DNA breaks will cause replicative stress, collapsed replication forks and single-ended DNA DSBs. Such lesions activate ATR, trigger phosphorylation of H2AX, visualised by γH2AX foci, and are resolved by HRR, visualised by RAD51 foci. Although low in the absence of exogenous DNA damage, γH2AX foci were modestly (27%, *p* = 0.059) higher and RAD51 foci were significantly (67%, *p* = 0.002) higher in Fus-1 cells than M059J cells (Table 2), indicative of higher levels of replicative stress.

**Table 2:** Replication stress in untreated M059J and Fus-1 cells

	M059J	Fus-1	*p*
γH2AX	5.12 ± 0.39 (292)	6.49 ± 0.66 (215)	0.059
RAD51	1.36 ± 0.15 (292)	2.27 ± 0.28 (215)	0.002

Data are mean ± standard error of the number of foci per nucleus (with the number of individual nuclei) measured in two independent experiments. P values were generated comparing foci in M059J with Fus-1 cells by unpaired t-test.

**Figure 4:**
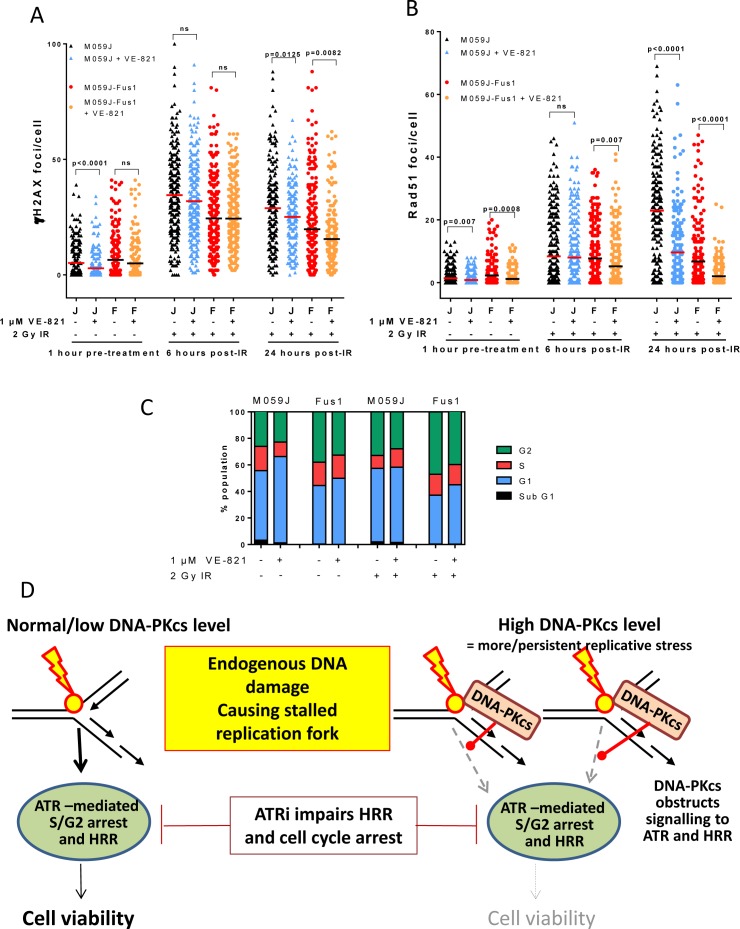
Effect of VE-821 on IR-induced DNA damage, HRR, cell cycle arrest and proposed mechanism in DNA-PKcs deficient and over-expressing cells **A.** γH2AX foci in M059J (J) and M059J-Fus-1 (F) treated with VE-821 (1 μM) and before or after exposure to 2 Gy IR as shown. **B.** RAD51 foci in M059J (J) and M059J-Fus-1 (F) treated with VE-821 (1 μM) and before or after exposure to 2 Gy IR as shown. **C.** Cell cycle profiles of M059J and M059J-Fus-1 exposed to VE-821 (1 μM) and 2 Gy IR as shown, measured after 24 hr. (Flow cytometry histograms shown in Supplementary Figure S5). **D.** Potential mechanism: In cells with low/normal levels of DNA-PKcs endogenous DNA damage (e.g. ROS-induced SS breaks) that cause stalling of the replication fork signal successfully to ATR to promote HRR and cell cycle arrest, ATR inhibition hampers this process but not sufficiently to seriously impact on cell viability. However, in cells expressing high levels of DNA-PKcs, this may compete with ATR signalling resulting in reduced HRR, if cells are also exposed to a DNA-PK inhibitor this will inhibit autophosphorylation and dissociation so may trap DNA-PKcs on the DNA. The postulated attenuation of signalling to HRR via DNA-PKcs obstruction combined with ATR inhibition may be the mechanism underlying the significantly compromised cell viability.

We previously showed that the ATR inhibitor NU6027 suppressed RAD51 focus formation, indicating that ATR inhibition led to an impairment of HRR [9]. To determine if VE-821 also suppressed HRR and to assess the impact of DNA-PKcs on HRR we measured γH2AX and RAD51 foci in M059J and Fus-1 cells exposed to 2 Gy ionising radiation (IR) in the presence or absence of VE-821 (Figure 4). IR was selected as the DNA damaging agent because a) it is effective and commonly used in cancer therapy, b) it reliably induces G2 accumulation in M059J and Fus-1 cells allowing direct comparison with the cell cycle data and c) published data indicates 1 μM VE-821 is a potent radiosensitiser [25] Interpretation of γH2AX data (Figure 4A) is somewhat complicated by the fact that DNA-PKcs is the major kinase responsible for H2AX phosphorylation immediately after IR. Previous work in our lab indicates that NHEJ is responsible for the immediate rapid DSB repair phase after IR and that the kinetics of resolution of γH2AX foci closely follows that of the resolution of DSB by neutral comet assay [20]. We selected 6 and 24 hr as suitable time-points to measure γH2AX foci as at these time-points they closely reflect DSB measurements. As such, the lower levels of γH2AX foci in Fus-1 cells at 6 and 24 hr may reflect the more rapid repair, resulting in faster disappearance of γH2AX foci. VE-821 caused a reduction in γH2AX foci at 24 hr, which may reflect an impairment of ATR-mediated phosphorylation of H2AX as IR-induced SS breaks encounter the replication forks.

Interestingly, VE-821 alone significantly suppressed RAD51 focus formation in unirradiated DNA-PKcs proficient and deficient cells, but the suppression was more profound in Fus-1 cells than in the M059J cells (Figure 4B, Table 3) *suggesting VE-821 impairs HRR-mediated repair of endogenous DNA damage/replication stress to a greater extent in DNA-PKcs overexpressing cells*. There were >3x more RAD51 foci in M059J cells than in Fus-1 cells (*p* < 0.0001) 24 hr after IR, potentially indicating that DNA-PKcs suppresses HRR or that DNA breaks had been repaired by the NHEJ pathway. DNA-PKcs may also suppress ATR activity as the phosphorylation of CHK1 after IR and gemcitabine was lower in Fus-1 than M059J cells (Supplementary Figures S1 and S2). VE-821 significantly (*p* < 0.0001) reduced RAD51 focus formation 24 hr post-IR in both cell lines, and, as in the absence of exogenous damage, a more profound suppression was observed in the Fus-1 cells (3.3-fold) than in the M059J cells (2.4-fold). *Cumulatively, these data suggest that VE-821 suppresses HRR to a greater extent in DNA-PKcs proficient Fus-1 cells, consistent with DNA-PK_CS_ proficient cells being more sensitive to VE-821-induced cytotoxicity.*

**Table 3:** RAD51 foci pooled data

Treatment	RAD51 foci/cell	
	M059J	Fus-1
Control	1.36 ± 0.15	2.27 ±0.28
10 μM VE-821	0.85 ± 0.11 (*p* = 0.007)	1.16 ± 0.16 (p=0.0008)
IR + 6 hr control	8.37 ± 0.69	7.72 ± 0.58
IR + 6 hr 1 μM VE-821	8.01 ± 0.61 (*p* = 0.69, NS)	5.18 ± 0.47 (p=0.0007)
IR + 24 hr control	22.94 ± 1.09	6.72 ± 0.69
IR + 24 hr 1 μM VE-821	9.6 ± 0.82 (*p*<0.0001)	2.02 ± 0.84 (p<0.0001)

Data are mean and standard deviation from 2 or more independent experiments where > 100 nuclei were evaluated. Significance is given for the effect of VE-821 by comparison with control or contemporaneously irradiated cells in the absence of VE-821

### VE-821 abrogates G2 arrest in both M059J and Fus-1 cells

We next sought to determine if the greater cytotoxicity of VE-821 in the DNA-PKcs expressing cells was due to a differential effect on cell cycle distribution. In parallel experiments to those measuring HRR, IR caused G2 accumulation, which was almost completely abrogated by 1 μM VE-821 in both cell lines, consistent with its inhibition of ATR signalling to the G2 checkpoint (Figure 4C) as shown previously in other cell lines [21].

## DISCUSSION

Clinical studies with molecularly targeted agents show that stratification of patients based on the molecular pathology of their tumour is critical for success [22]. ATR has emerged as an interesting novel target and two ATR inhibitors are being tested clinically. The aim of the work presented here was to identify molecular determinants of sensitivity to ATR inhibitors for patient stratification. Since dysregulation of the DNA damage response (DDR) is common in cancer, and there is precedent for DDR defects to confer sensitivity to DDR inhibitors (HRR defects and PARP inhibitors)), we focussed on DDR defects as potential determinants of sensitivity to single agent ATR inhibition.

BER and HRR are complementary pathways in the repair of endogenous DNA damage. It is clear that VE-821 impairs HRR since it causes a profound suppression of RAD51 focus formation after IR (Figure 4B). By analogy with the synthetic lethality of PARPi (which suppresses BER) in HRR-defective cells, the reciprocal synthetic lethality is also predicted, where inhibition of HRR by VE-821 is synthetically lethal with defects in BER as indicated by the hypersensitivity of the BER-defective EM9 cells confirming our previous results with another ATR inhibitor, NU6027 [9]. BER defects have been reported widely in a variety of cancer types (reviewed in [23]) and selection of patients based on tumour BER status for ATR inhibitor monotherapy may be a useful therapeutic manoeuvre.

The hypersensitivity of ATM-defective cells was also not unexpected because VE-821 had previously been shown to be cytotoxic to cells in which ATM had been knocked down [11]. Moreover, recent data, reported in abstract form only, indicate that the ATR inhibitor AZD-6738 was synthetically lethal in ATM-defective CLL cells from patients [12]. ATM is thought to contribute to HRR [24] and we found that other HRR defects also conferred sensitivity: Irs1SF cells defective in XRCC3 (a RAD51 related protein) were the most sensitive to VE-821 and BRCA2-mutant cells showed a similar level of sensitivity to the ATM mutants. Thus, although ATR inhibitors are thought to principally modulate HRR, synthetic lethality can be achieved in cells that already have defects in components of HRR, indicating that epistasis is not an issue. This is consistent with the observation that defects in the ATR signalling pathway conferred substantial synthetic lethality with ATR and CHK1 inhibitors and analogous to recent data showing that *XRCC1*^−/−^ cells are hypersensitive to PARP inhibitors [25]. These data suggest that ATR inhibitors may have activity in several tumour types because HRR defects are common in cancer, not just those associated with BRCA1/2 mutations [26]. The potential could be very broad indeed as hypoxia, a common occurrence in tumours that promotes a more aggressive phenotype, actually causes a downregulation of HRR proteins that is exploitable by “contextual synthetic lethality” [2]. Indeed, this may underlie the cytotoxicity and radiosensitisation by VE-821 in hypoxic cells [27].

In contrast to reports that ERCC1, a nuclease in the NER pathway was synthetically lethal with ATR inhibition [11], dysfunction of ERCC2 (XPD), a helicase upstream of ERCC1 in the NER pathway, only conferred a modest sensitivity to VE-821. ERCC1 also participates in multiple DNA repair pathways, including cross-link repair and HRR [28] that may contribute to the sensitivity to VE-821. Therefore, not all DDR defects confer substantial sensitivity to ATR inhibitors.

Our most surprising observation was that cells defective in DNA-PKcs were not sensitive to VE-821 but VE-821 was profoundly cytotoxic to cells that over-expressed DNA-PKcs. The initial observation in CHO cells was confirmed in two isogenic pairs of human cancer cell lines. The kinase function of DNA-PKcs was not responsible because co-exposure to the DNA-PK inhibitor, NU7441 increased, rather than decreased, sensitivity to VE-821. Similarly, the Ku80-defective cells were sensitive rather than resistant to VE-821 suggesting that NHEJ function was not responsible. However, it should be noted that Ku80 has other functions besides NHEJ, such as telomere maintenance [29, 30] a possible role in BER [31] and it has been implicated in HRR through its interaction with BRCA1 [32]. Since both BER and HRR defects confer sensitivity to VE-821 the sensitivity of Ku80 defective cells may reflect these alternative functions.

Overexpression of cMYC has previously been shown to cause replicative stress and confer sensitivity to CHK1 inhibitors and ATR knockdown [15, 33]. Since correction of the DNA-PKcs defect in Fus-1 cells involves amplifying chromosome 8 and that the cMYC gene is also on chromosome 8 we investigated if cMYC was implicated in the VE-821-sensitivity of DNA-PKcs over-expressing cells. Fus-1 cells did have significantly higher levels of cMYC and γH2AX and RAD51 foci, suggesting a higher level of replication stress (Figure 4A/B). However, when tested in another cell background, without manipulation of DNA-PK_CS_, cMYC over-expressing cells were resistant rather than sensitive to VE-821 indicating that it is the elevated DNA-PKcs rather than cMYC that dictates VE-821 sensitivity. We hypothesise that DNA-PKcs over-expression, which is common in cancer [34], causes genomic instability by competition with HRR and needs to be balanced by an increase in ATR to allow the growth of the tumour. Indeed, data mining confirms that, in gliomas at least, there is a strong correlation between *ATR* and *PRKDC* transcripts. The higher level of RAD51 foci in Fus-1 cells compared to M059J cells was accompanied by a higher G2 fraction that may also indicate increased replication stress and dependence on G2 checkpoint signalling. VE-821 caused a much greater reduction in RAD51 and slightly greater reduction in the G2 fraction in untreated Fus-1 cells. This suggests that in DNA-PKcs overexpressing cells VE-821 has a greater impact on HRR and checkpoint activation in response to endogenous damage. Following exposure to IR there was a greater increase in RAD51 foci in M059J cells compared to Fus-1 cells, suggesting that HRR was more active in DNA-PKcs defective cells. Remarkably, VE-821 caused a greater suppression of RAD51 foci in the Fus-1 cells, confirming its greater impact on HRR in the DNA-PKcs expressing cells. Several studies suggest that NHEJ competes with HRR in the response to DNA DSBs (reviewed in [35]) and indeed the deletion of 53BP1 or DNA-PKcs can restore HRR function in BRCA mutant cells [36]. Our data are largely in agreement with these observations but suggest that different components of NHEJ are more important for this competition than others. Thus, Ku80 and the NHEJ pathway *per se* may not be critical, but DNA-PKcs may hamper HRR. Since HRR is a high fidelity pathway and specifically associated with stalled replication forks (likely to be the most abundant endogenous DS lesions) it is the pathway principally used to maintain viability in the absence of exogenous DNA damage. Indeed, whereas HRR is critical for genomic stability, NHEJ, which is more error-prone, has been implicated in promoting genomic instability and cancer development [35].

In contrast to reports that DNA-PK inhibition (with NU7441) also restored HRR function and resistance to PARPi in BRCA mutant cells [36], we found that NU7441 actually increased sensitivity to VE-821. DNA-PKcs autophosphorylation is thought to be necessary for its dissociation from DNA for the NHEJ pathway to complete DSB repair. Based on our observations we speculate that increased DNA-PKcs levels increases the chance of binding to DNA DSB potentially obstructing the recruitment of some components of the HRR machinery (Figure 4D). We further speculate that this potential impairment of HRR causes hypersensitivity to VE-821, similar to that experienced by HRR defective cells. The observation that NU7441 further sensitised Fus-1 cells to VE-821 may suggest that inhibition of the kinase activity could hinder DNA-PKcs dissociation and hence further impair recruitment of the HRR machinery. This would be analogous to PARP inhibitors which, by preventing PARP-1 auto-ADP-ribosylation and dissociation from DNA breaks, have a more profound effect on their repair than does depletion of the enzyme [37]. Similarly, ATM inhibition is more detrimental than ATM knockdown, through inhibiting ATM dissociation from the DNA thereby causing a physical impediment [8, 38]. Further investigations would be necessary to determine the therapeutic potential of DNA-PKcs and ATR inhibitor combinations.

HRR defects due to BRCA mutations are already known to confer sensitivity to both conventional cytotoxic agents (especially cisplatin) and molecularly targeted agents (PARPi). Our observations that BER defects and high levels of DNA-PKcs confer sensitivity to ATR inhibition are therefore novel and exciting. Whilst the sensitivity of cells with these imbalances in the DDR to ATR inhibition may not be quite as striking as reported originally for BRCA mutations and PARP, they are similar to our previous determination of sensitivities of HRR-defective human cancer cells to PARP inhibition [39] and other synthetic lethalities with ATM and ATR inhibition [40] and reviewed in [41]). As stated above, both BER defects and high NHEJ can be sufficient to promote genomic instability leading to cancer formation. Several cancers have defects in BER and/or overexpress DNA-PKcs [23, 34]. Over-expression of DNA-PKcs may be linked to disease progression, and our own data indicate that this is the case in liver cancer [42]. The data presented here indicate that BER defects and DNA-PKcs overexpression are exploitable with the ATR inhibitor, VE-821 and may prove to be predictive biomarkers for personalised medicine with the ATR inhibitors that are in clinical trials (https://www.clinicaltrials.gov/ct2/results?term=ATR+inhibitor&pg=1).

## MATERIALS AND METHODS

### Chemicals and reagents

Routine chemicals and reagents were obtained from Sigma Aldrich (Poole, UK) unless otherwise stated. The ATR inhibitor VE-821 (Vertex Pharmaceuticals (Europe) Ltd Abingdon, UK) VE-821 and the DNA-PK inhibitor NU7441 (kind gift from Celine Cano, Newcastle University, UK) were dissolved in DMSO and stored at −20°C.

### Cell lines

Parental Chinese hamster ovary (CHO) cells (AA8) and their repair-defective derivatives: Irs1SF (XRCC3 defective: BER), xrs6 (Ku80 defective: NHEJ), V3 (DNA-PK_CS_ defective: NHEJ) and V3-YAC cells (V3 cells complemented with human DNA-PK_CS_) were a kind gift from Professor Penny Jeggo (Sussex University, Brighton, UK). EM9 (XRCC1 defective), and UV5 (ERCC2 defective) were obtained from American Type Culture Collection (ATCC, VA, USA). All CHO cells were grown in RPMI 1640 media supplemented with 10% FBS. V3-YAC cells were grown in medium with G418 (400 μg/ml).

Parental Chinese hamster lung fibroblast (CHL) cells (V79) and their repair-defective derivatives V-E5 (ATM defective) were a kind gift from Professor Srinivasan Madhusudan (Nottingham University, UK). VC8 (BRCA2 defective;HRR) V-C8-B2 (VC8 cells complemented with human BRCA2 and V-C8 PiR (VC8 clone spontaneously resistant to PARP inhibitors, [43]), kindly provided by Professor Thomas Helleday (Karolinska, Stockholm) were grown in Dulbecco's Modified Eagle's Medium (DMEM) supplemented with 10% FBS. V-C8 B2 cells were grown in medium with G418 (400 μg/ml).

MCF-10A cells were purchased from American Type Culture Collection (ATCC, VA, USA) and authenticated by SNP 6.0 and published karyotype data, [44, 45]. Myc-MCF-10A cells were generated by exposing cells to 5 Gy fractionated doses of ionising-radiation to a cumulative dose of 80 Gy as previously described in [1]. MCF-10A and Myc-MCF-10A cells were maintained in Dulbecco's Modified Eagle's Medium: Nutrient Mixture F-12 (DMEM-F12) containing 5% (v/v) horse serum, 20 ng/ml epidermal growth factor, 0.5 μg/ml hydrocortisone, 100 ng/ml cholera toxin and 10 μg/ml insulin.

M059J, DNA-PKcs-deficient human glioblastoma cells [17], were grown in DMEM supplemented with 10% FBS. M059J-Fus-1 (M059J transfected with a portion of chromosome 8 carrying the DNA-PKcs gene; [16]) cells were cultured in full media with 400 μg/ml G418. Cells were authenticated by STR profiling (LGC Standards, Teddington UK). Knockdown models were generated by transfection of OSEC2 cells [46] with viral particles made using Mission lentiviral packaging mix (Sigma-Aldrich) as per manufacturer's protocol. OSEC shOT (containing MISSION^®^ pLKO.1-puro Non-Target shRNA Control Plasmid DNA) or OSEC shDNA PK (containing PRKDC MISSION shRNA Lentiviral Transduction Particles;

**Sequence:** CCGGGCAGATAGAAAGCATTACATTCTCGAGAATGTAATGCTTTCTATCTGCTTTTT; TRCN0000006255) were generated. Following transfection, cells stably expressing the appropriate shRNA constructs were selected for by culturing in HEPES modified RPMI-1640 containing 10% FBS, 100 unit/ml penicillin/streptomycin and 400 μg/mL G418) and incubated at 33°C with 5% CO_2_.

### Cytotoxicity assays

Exponentially growing cells were counted and seeded at known low densities into 6-well tissue culture plates and allowed to adhere overnight. Cells were treated with a range of VE-821 concentrations in 0.1% DMSO in complete medium for 24 hours and control cells were exposed to 0.1% DMSO. Surviving cells were allowed to grow in fresh drug-free media for 1-2 weeks to form colonies (> 30 cells) prior to fixation (75% methanol, 25% glacial acetic acid), staining (1% crystal violet) and counting. Survival was calculated by reference to the DMSO controls.

### Western blotting

Samples of exponentially growing cells were harvested by brief trypsinisation and protein extracted by re-suspending the cell pellet in Phosphosafe Extraction Reagent (Merck, EMD Millipore, MA, USA) containing protease inhibitor cocktail at room temperature for 10 minutes followed by brief sonication. 30 μg of protein for each sample was analysed by SDS PAGE and Western blotting unless otherwise stated. Actin was detected using a 1:10,000 dilution of THE™ beta Actin Antibody (Genscript, NJ, USA), cMYC using a 1:5000 dilution of Anti-cMYC [Y69] antibody (Abcam, Cambridge, UK), ATR using a 1:300 dilution of ATR Antibody N-19 (Santa Cruz Biotechnology Inc, TX, USA) and DNA-PK using a 1:300 dilution of DNA-PKcs Antibody (H-163) (Santa Cruz Biotechnology Inc, TX, USA) all were incubated overnight at 4°C. HRP-conjugated secondary antibodies; Goat anti-rabbit IgG-HRP, Goat anti-mouse IgG-HRP (Dako, Cambridge, UK) and Donkey anti-goat IgG-HRP (Santa Cruz Biotechnology Inc, TX, USA) were all used at a 1:1000 dilution and incubated for 1 hour at room temperature. Image capture and analysis were carried out using the Fuji LAS-300 Image Analyser System (Raytek, Sheffield UK).

### cMYC stability

Exponentially growing M059J and M059J-Fus-1 cells were treated with 1 μM NU7441 and 40 μg/mL cyclohexamide and incubated for 0, 30, 60, 120 or 240 minutes as indicated prior to protein extraction and western blotting as described above.

### DNA damage and repair assays

Cells seeded onto coverslips at a density of 0.5 × 10^5^ cells/ml were allowed to adhere for 24 hr then treated with VE-821 and/or NU7441 at the concentrations stated for 1 hour prior to 2 Gy X-ray irradiation. Cells were then incubated for 6 and 24 hours prior to fixing with ice cold methanol at −20°C. Immunofluorescence microscopy for phospho-Histone H2AX (Ser 139; γH2AX) and RAD51 foci was carried out as described previously [47]. The number of γH2AX and RAD51 foci was determined in > 50 cells per condition using ImageJ software and a custom macro [48].

### Cell cycle analysis by flow cytometry

Approximately 1 million cells were seeded into tissue culture dishes and allowed to adhere overnight. Cells were treated with VE-821 and/or NU7441 at the concentrations stated and/or 2 Gy X-irradiation. Where irradiation was used cells were pre-treated with VE-821/NU7441 for 1 hour. After 24 hours, cells were trypsinised and fixed in ice cold methanol stored overnight at −20°C then washed in PBS and stained using PBS containing 200 μg/ml propidium iodide and 200 μg/ml RNAase A. Following incubation for at least 30 minutes in subdued light, cells were analysed using a FACSCalibur Flow cytometer (BD Biosciences, San Jose, CA, USA) using a 488 laser. At least 20,000 events were counted and analysed using Cyflogic analysis software (Cyflo Ltd, Turku, Finland).

### Mining of publically available mRNA expression data

mRNA expression profiles were obtained from the publically available NCBI Gene Expression Omnibus (GEO). mRNA expression data from the GEO dataset GSE4290 was used and mean *ATR* and *PRKDC* (gene encoding DNA-PK_CS_) mRNA levels, measured by multiple probes on the Affymetrix Human Genome U133 Plus 2.0 Array, were normalised to that of the most consistently expressed housekeeping gene; *HPRT1*. Samples where information from appropriate probes was available included 23 normal (epilepsy patients) samples, 26 astrocytomas, 77 glioblastomas and 50 oligodendrogliomas. mRNA expression was compared and correlated using GraphPad Prism version 6.0.

### Statistical analysis

Statistical analysis was performed using GraphPad Prism 6.0 (San Diego, CA, USA). Paired or unpaired t-tests were performed as indicated and, where clonogenic survival was assessed, groups were compared using a 2-way ANOVA with application of a Bonferroni correction. Differences were deemed significant when *p* < 0.05.

## SUPPLEMENTARY MATERIAL FIGURES


