# Supplementary material for: Common cancer-associated imbalances in the DNA damage response confer sensitivity to single agent ATR inhibition
Source: Oncotarget. 2015 Oct 15;6(32):32396–409. doi: 10.18632/oncotarget.6136 (PMC4741701; doi:10.18632/oncotarget.6136)
Supplement: Supplementary file 1 [file oncotarget-06-32396-s001.pdf]

Common cancer-associated imbalances in the DNA damage response confer sensitivity to single agent ATR inhibition

Supplementary Material

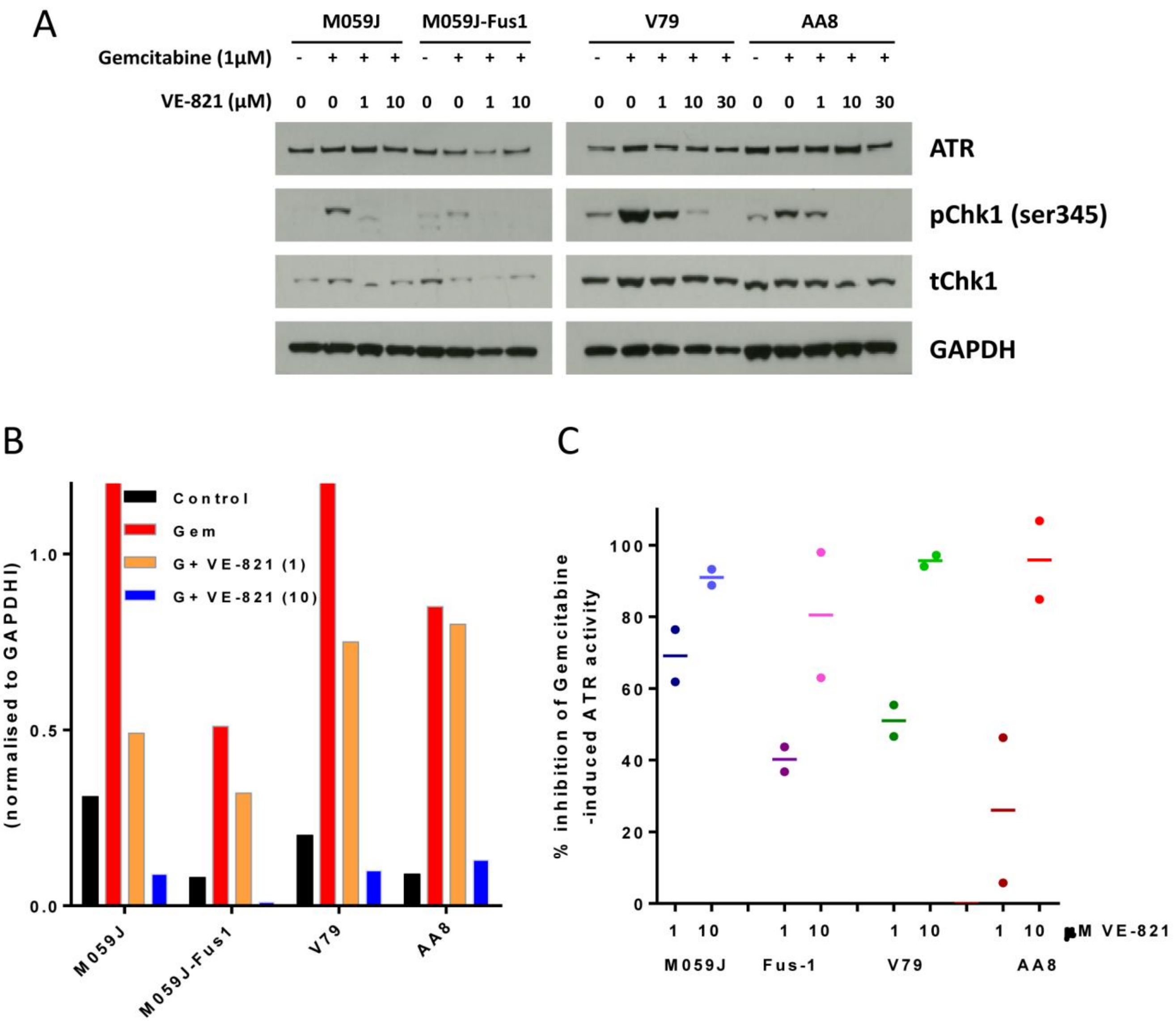

Supplementary Fig S1: VE-821 inhibits ATR activity to a similar extent in a range of human and hamster cells. Inhibition of ATR (Chk1 phosphorylation) by VE-821 at concentrations used in the cytotoxicity assays. A: western blot, lower panel pChk1s^{345} normalised to GAPDH. Cells were exposed to 1 µM Gemcitabine ± VE-821 for 1 hr. B. pChk1s^{345} levels normalised to actin for a single experiment (G, Gem = gemcitabine), C % inhibition Gemcitabine-induced CHK1 phosphorylation in 2 replicate experiments (data points represent the 2 experiments. VE-821 at 1 µM inhibited ATR by approximately 50% (25-75%) in all cell lines and 10 µM inhibited ATR by around 90%. There was no detectable pChk1s^{345} at 30 µM VE-821

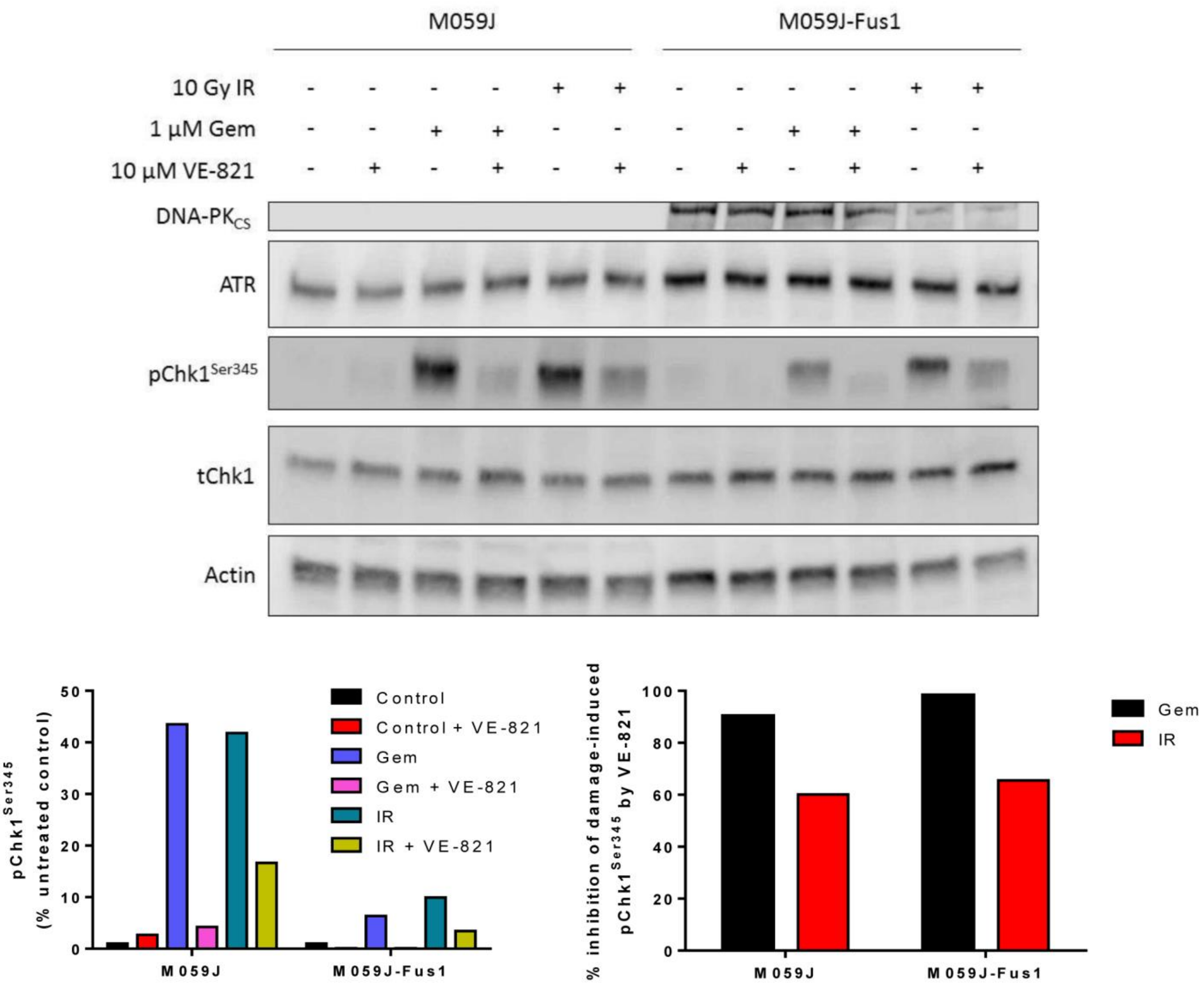

Supplementary Fig S2: VE-821 inhibits both IR and gemcitabine-induced ATR activity to a similar extent in M059J and Fus-1 cells. Fiona to supply experimental details

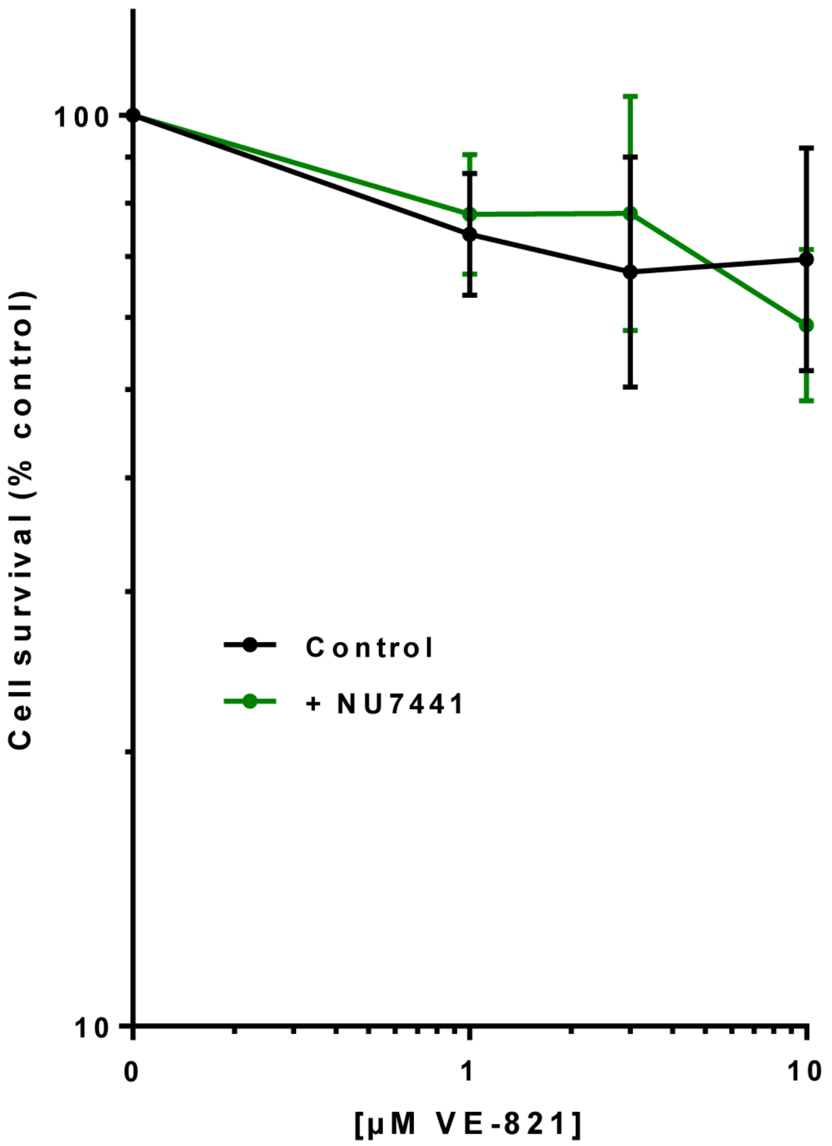

Supplementary Fig S3: NU7441 does not increase the cytotoxicity of VE-821 in M059J cells. Cells were exposed to varying concentrations of VE-821 for 24 hr then allowed to form colonies in drug free medium. Data are mean and standard deviation of 3 independent experiments with duplicate samples/experiment

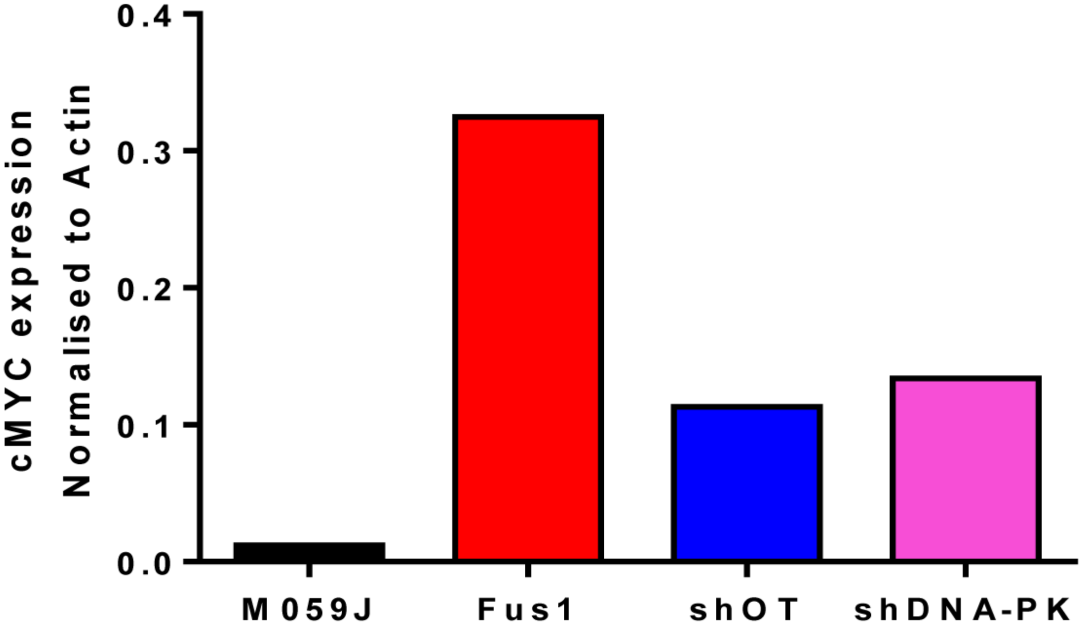

Supplementary Fig S4: cMYC expression in M059J, M059J-Fus-1, OSEC2 shOT and OSEC2 shDNA-PK cells . Replicate experimental data (analogous to that shown in Figure 3A) cMyc protein levels normalised to actin.

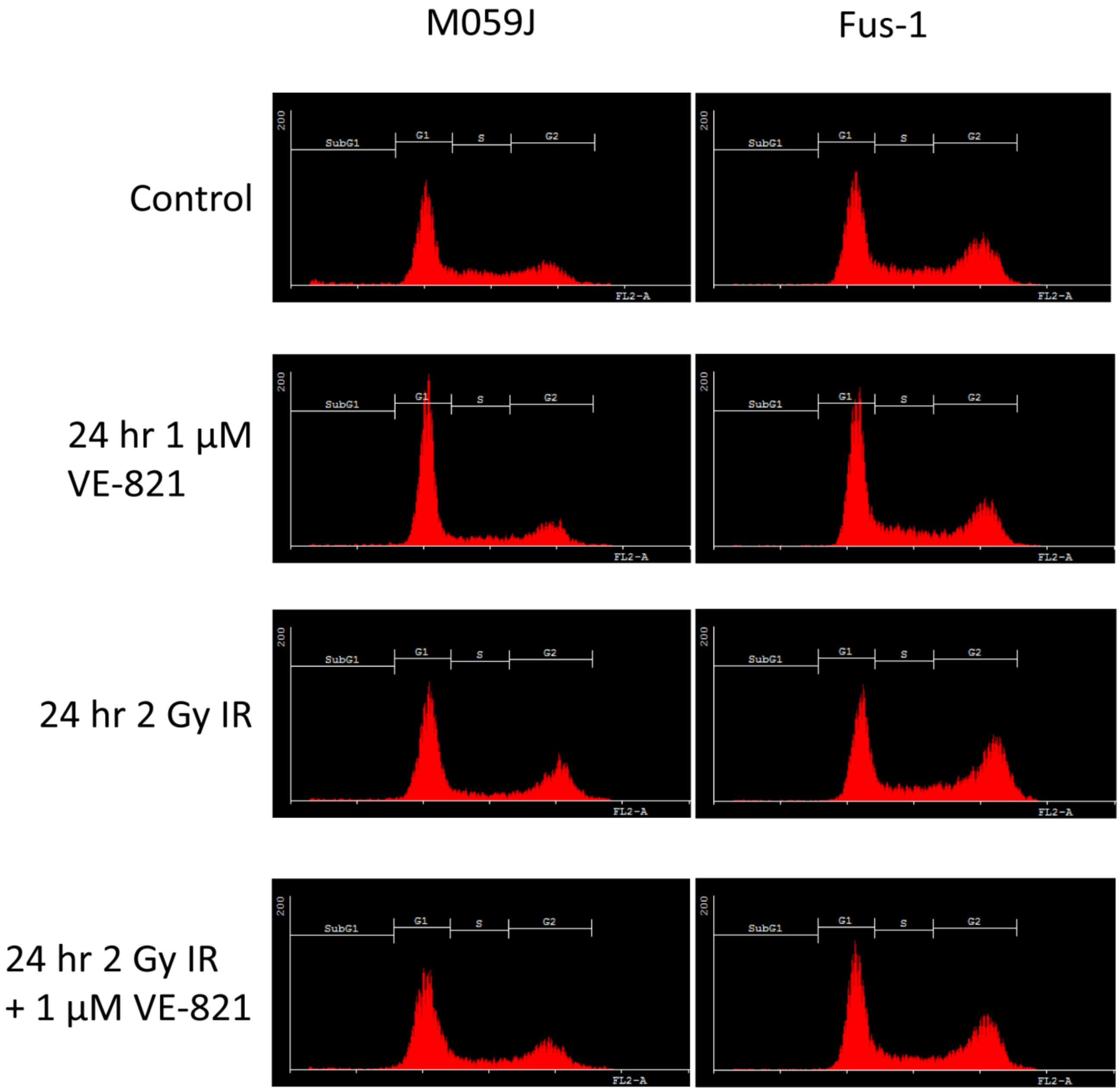

Supplementary Fig S5: Cell cycle profiles of M059J and Fus-2 cells following exposure to IR ± VE821
